# Sensitisation pattern to birch pollen allergen components in oral allergy syndrome to Rosaceae fruits in patients with spring pollinosis from an East European Sylvosteppe area with low density forests

**DOI:** 10.1186/2045-7022-4-S2-P47

**Published:** 2014-03-17

**Authors:** Mariana Vieru, Florin-Dan Popescu, Adriana Tudose, Nicolae Dumitrescu

**Affiliations:** 1 University of Medicine and Pharmacy"Carol Davila", Hospital "Nicolae Malaxa", Department of Allergology and Clinical Immunology, Bucharest, Romania; 2 University of Medicine and Pharmacy"Carol Davila", Hospital"Nicolae Malaxa", Department of Allergology and Clinical Immunology, Bucharest, Romania; 3 Hospital "Nicolae Malaxa", Department of Allergology and Clinical Immunology, Bucharest, Romania

## Background

The cross-reactive molecular allergen components involved in birch pollen-food syndrome depend of the type of food, severity of the food allergic reaction, patient age and the geographic environment, including climate and presence of Fagales-endemic areas.

## Methods

We evaluated subjects from Southern Romania, a Central European region with temperate continental climate with submediterranean and humid subtropical influences, where sensitization to Betulaceae pollen is less important in pollinosis compared with that to grass or weed pollen. We selected adult patients from the region of sylvosteppe with low density forests dominated by deciduous species, presenting symptoms of rhinoconjunctivitis in March to May and oral allergy syndrome to fresh Rosaceae fruits, allergic reactions in the mouth and throat without systemic symptoms, and having positive skin prick tests to Betulaceae tree pollen and positive prick-to-prick skin tests with fresh intact and unpeeled apple, pear, apricot, peach, cherry or plum. Because allergen components present in Fagales tree pollen and Rosaceae fruits are PR-10 proteins, profilins and isoflavone reductases, we measured the serum levels of specific IgE to European birch pollen and to recombinant allergen components Bet v1 (birch major PR-10 allergen with ribonuclease activity), Bet v2 (birch-pollen profilin), and Bet v6 (birch-pollen isoflavone reductase), using a multiparameter immunoblot test system based on single purified allergen components (SPAC 1).

## Results

Only a case series of four adult spring pollinosis patients with oral allergy syndrome to fresh apple, pear, peach or plum, was detected, and all had specific IgE against rBet v 1 (genuine sensitization to birch pollen). This is important because Bet v 1 homologues are also found in Rosaceae fruits (PR-10 proteins with 50-60% identity to Bet v 1). Measured levels of serum specific IgE to rBet v 1 varied between 30 to 49 kU/L. Specific IgE againts rBet v 2, profilin sensitization marker, rBet v 4, polcalcin sensitisation marker, and rBet v 6, assessing sensitization to birch pollen isoflavone reductase, were not detected.

## Conclusion

Bet v 1 sensitization is associated to concomitant birch pollen rhinoconjunctivitis and oral allergy syndrome to Rosaceae fruits in patients from the Southern Romania sylvosteppe area with low density forests.

